# The Hsp90β Isoform: An Attractive Target for Drug Development

**DOI:** 10.1002/med.22114

**Published:** 2025-04-28

**Authors:** Subhabrata Chaudhury, Terin D'Amico, Brian S. J. Blagg

**Affiliations:** ^1^ Department of Chemistry and Biochemistry, Warren Family Research Center for Drug Discovery and Development University of Notre Dame Notre Dame Indiana USA; ^2^ Department of Chemistry and Biochemistry University of Notre Dame Notre Dame Indiana USA

**Keywords:** drug discovery, Hsp90 beta, isoform selectivity, structure‐based drug design

## Abstract

The beta isoform of 90 kDa heat shock protein (Hsp90β) plays a critical role in maintaining cellular proteostasis by assisting in the folding and refolding of proteins, which is essential for both normal cellular function and stress response. It is constitutively expressed in mammalian cells, differentiating it from the inducible Hsp90α isoform. Hsp90β's involvement in diverse cellular processes, such as signal transduction, cell cycle control, and apoptosis, underscores its significant role in various diseases, including cancer and neurodegenerative disorders. The isoform‐specific functions of Hsp90β and its interaction with unique client proteins make it a promising target for therapeutic intervention, particularly in the development of selective inhibitors that avoid the adverse effects observed with pan‐Hsp90 inhibitors. This review delves into the structural and functional intricacies of Hsp90β, its role in disease, and the potential for selective drug development.

**Abbreviations**
Hsp90β: Heat shock protein 90 beta
IAP: Inhibitor of apoptosis protein
IC50: Half‐maximal inhibitory concentration
IL‐1β: Interleukin 1 beta
IL‐8: Interleukin 8
KD: Dissociation constant
LC: Laryngeal carcinoma
LPS: Lipopolysaccharide
LR: Linker region
LRP‐1: Lipoprotein receptor‐related protein 1
MAPK: Mitogen‐activated protein kinase
MD: Middle domain
MDR: Multidrug resistance
MEEVD: Met‐Glu‐Glu‐Val‐Asp (conserved Hsp90 sequence)
Mip‐90: Microtubule‐interacting protein 90
MM: Multiple myeloma
MMP3: Matrix metalloproteinase 3
MS: Multiple sclerosis
mHTT: Mutant huntingtin
NAFLD: Nonalcoholic fatty liver disease
NADPH: Nicotinamide adenine dinucleotide phosphate (reduced form)
NCI H23: National Cancer Institute human lung cancer cell line H23
NF‐κB: Nuclear factor kappa‐light‐chain‐enhancer of activated B cells
NO: Nitric oxide
NTD: N‐terminal domain
ODNs: Oligodeoxynucleotides
PDB: Protein Data Bank
PGP: P‐glycoprotein
PPARγ: Peroxisome proliferator‐activated receptor gamma
PRRSV: Porcine reproductive and respiratory syndrome virus
PTMs: Posttranslational modifications
RNA: Ribonucleic acid
ROS: Reactive oxygen species
SAR: Structure–activity relationship
Ser: Serine
SNX‐2112: Pan‐Hsp90 inhibitor compound
SNX‐5422: Orally available prodrug of SNX‐2112
TGFα: Transforming growth factor alpha
TGFβ1: Transforming growth factor beta 1
TPR: Tetratricopeptide repeat
TRAP1: Tumor necrosis factor receptor‐associated protein 1
VEGFR: Vascular endothelial growth factor receptor

## 1 Introduction

In 1962, Italian scientist Ferruccio Ritossa published seminal work, wherein he described the reversible activation of specific genes present in the salivary glands of *Drosophila busckii* that were overexpressed in response to heat, radiation (tritiated cytidine) or chemicals (2,4‐dinitrophenol and sodium salicylate) [1]. This phenomenon has been identified as an evolutionary conserved mechanism against stress in almost all living organisms, including humans, and termed the “heat shock response (HSR)” [2]. The overexpressed proteins that occur in response to the HSR are referred to as heat shock proteins and are categorized based on molecular weight and location [3, 4].

The 90 kDa heat shock proteins constitute 1–2% of normal cellular proteins and perform their duty as a chaperone to mitigate cellular stress by refolding denatured proteins back into their active conformation. The chaperoning activity coordinated by Hsp90 is important to both unstressed and normal cells, in which molecular chaperones maintain cellular proteostasis by folding newly synthesized polypeptides into their three‐dimensional conformations, as well as the refolding of denatured proteins back into their active form [5]. Hsp90 and its co‐chaperones interact with client protein substrates, many of which modulate signal transduction processes, and therefore, dysregulation of Hsp90 can contribute to a wide array of diseases including cancer, neurodegeneration, and viral infection amongst others [5, 6, 7, 8, 9, 10, 11].

Mammalian cells contain two cytosolic isoforms; inducible Hsp90α and constitutively expressed Hsp90β. Though primarily located in the cytosol, Hsp90α and Hsp90β can also be found to a lesser extent in the nucleus [12, 13, 14, 15]. In addition, ER localized 94‐kDa glucose‐regulated protein (Grp94) and mitochondrial tumor necrosis factor receptor‐associated protein 1 (TRAP1) are organelle specific isoforms of Hsp90 [5, 16, 17, 18, 19]. Although, the Hsp90 isoforms share more than 85% identity within the N‐terminal ATP binding pocket, their function and client protein substrates vary [19, 20, 21]. Approximately 400 client proteins, many of which are responsible for the growth and proliferation of cancer cells, are modulated by these Hsp90 isoforms [22, 23]. As a result, 19 pan‐Hsp90 inhibitors underwent clinical evaluation for the treatment of cancer, however various adverse effects, such as cardiotoxicity, gastrointestinal toxicity and/or ocular toxicity have prevented subsequent investigation with most of these inhibitors [24, 25, 26, 27].

In addition to intracellular functions, both Hsp90α and Hsp90β are secreted into the extracellular medium (plasma and serum). The corresponding secreted or extracellular Hsp90 isoforms are referred to as eHsp90α and eHsp90β respectively [28, 29, 30, 31, 32, 33, 34].

Hsp90α and β are highly homologous and exhibit ~85% overall sequence identity and > 95% identity within the N‐terminal ATP‐binding site, which is most likely the consequence of divergent evolution from a single gene [35, 36]. Regardless, they exhibit distinguishing functions. Hsp90α is highly inducible, while Hsp90β is constitutively expressed and participates in more common cellular processes, such as protein folding and stability, signaling pathways, and cell cycle regulation [37]. Although Hsp90β is constitutively expressed, it may also be upregulated with stress [38]. The translocation of Hsp90β from the cytosol to the cell membrane occurs while interacting with the NADPH oxidase complex via infected gastric epithelial cells [39].

It has been proposed that the plasma membrane receptor protein, cluster of differentiation 91 (CD91), interacts with exosome Hsp90α through its middle and charged linker domain while promoting the epidermal and dermal migration of cells via the transforming growth factor α (TGFα) signaling pathway [40]. The eHsp90α mediated pro‐motility activity that occurs during wound healing and cancer is due to a 115 –amino acid containing middle and charged linker binding domain, referred as “F‐5” [41]. The 115‐aa secreted fragment of Hsp90α demonstrated acute and diabetic would closure properties in mice by recruiting both epidermal and dermal cells, promoting dermal cell migration, and overriding the inhibitory effects of hyperglycemia on cell migration in diabetes [42]. Experimental evidence suggests that the natural product, gambogic acid (GBA) and its analog DAP‐19, bind preferentially to this middle domain of Hsp90β [41].

Sequencing and mapping studies of the human genome have indicated that all four Hsp90 isoforms result from successive gene duplications, wherein genetic duplication of Hsp90α and Hsp90β occurred more recently as compared to other Hsp90 isoforms, accounting for the greater identity shared among these paralogs. [16, 35, 36, 43, 44, 45, 46, 47, 48].

In this review, the rationale and development of cytosolic Hsp90β‐selective inhibitors will be discussed along with β‐specific disease implications.

## 2 Hsp90 Structure and Chaperone Cycle

**Figure 1:**
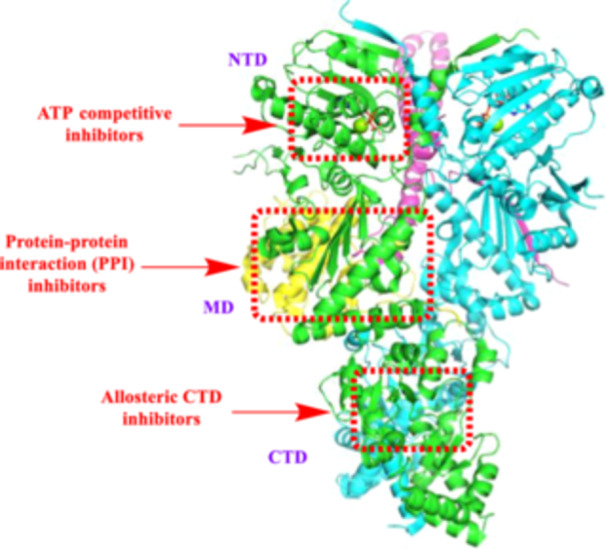
Structure of the Hsp90β homodimer, associated domains and locations for class of inhibitors. (PDB Code: 5FWK). [Color figure can be viewed at wileyonlinelibrary.com]

Hsp90's functional versatility stems from its complex structure, which comprises three major domains (Figure 1): the N‐terminal domain (NTD), middle domain (MD), and C‐terminal domain (CTD). Additionally, eukaryotic Hsp90 uniquely contains a fourth domain called the charged linker region (LR) between the NTD and MD. These domains orchestrate Hsp90's chaperone activity by coordinating client protein binding, ATP hydrolysis, and conformational changes. Here, we delve into the structural intricacies of Hsp90, drawing insights from seminal studies.

The NTD (~25 kDa) is characterized by a mixed α/β fold, consisting of a central β‐sheet surrounded by α‐helices. It houses the ATP‐binding pocket, critical for the chaperone's activity, and serves as a platform for dimerization. Structural studies, including X‐ray crystallography and NMR spectroscopy, have provided detailed insights into the ATP‐binding site within the NTD, revealing conserved motifs essential for nucleotide binding and hydrolysis [49, 50].

The LR consists of a disordered region between the NTD and MD [51, 52]. The LR in Hsp90 is a flexible, highly charged amino acid sequence connecting the NTD and MD. It is rich in acidic and basic residues, giving it a strong net charge, and serves as a flexible hinge, allowing the domains to move relative to each other. This linker is particularly notable for its variability in length and sequence among Hsp90 isoforms and across species, which can influence the chaperone's structural dynamics and client‐binding specificity. The charged linker also plays a regulatory role, facilitating interactions with various co‐chaperones, such as Cdc37 and Aha1, which modulate Hsp90's ATPase activity and stabilize client proteins.

The MD of Hsp90 (~35 kDa) acts as a flexible linker connecting the NTD and CTD. Structurally, it adopts a predominantly α‐helical fold and contains conserved motifs, which are crucial for interaction with client proteins and co‐chaperones such as Hsp70 and Hop [53]. Structural elucidation of the MD has highlighted its role in mediating inter‐domain communication and coordinating co‐chaperone recruitment, thereby modulating Hsp90's chaperone activity [43].

The CTD of Hsp90 (~12 kDa) is characterized by a highly charged surface enriched with acidic residues. Structurally, it adopts a β‐sandwich fold stabilized by multiple disulfide bonds [54]. This domain contains several binding regions; a nucleotide binding site, the region between the MD and dimerization interface and a pentapeptide motif (MEEVD) at the termini [55]. The MEEVD (Met‐Glu‐Glu‐Val‐Asp) sequence is a conserved C‐terminal motif found only in eukaryotic Hsp90 that binds to TPR‐domain (tetratricopeptide‐containing repeats). CTD serves as the primary site for client protein recognition and interaction with co‐chaperones, playing a pivotal role in the folding and stabilization of client proteins [25].

Interactions between the NTD, MD, and CTD govern Hsp90's structural dynamics and functional activity. Flexibility within the linker regions connecting these domains allows for conformational rearrangements essential for client protein binding and ATP‐dependent conformational changes. Cryo‐electron microscopy (cryo‐EM) studies have provided valuable insights into the dynamic nature of Hsp90, capturing different conformational states of the chaperone complex during the chaperone cycle [53].

Heat shock protein 90 (Hsp90) is a master regulator of proteostasis, exerting its chaperone activity to shepherd an array of client proteins through the intricate process of protein folding and maturation. The chaperone activity of Hsp90 transcends to late stage protein folding of client proteins into functional forms, encompassing regulatory functions in signal transduction, transcriptional regulation, and protein degradation pathways [11, 56, 57, 58]. The dynamic interplay between Hsp90 and its client proteins, the molecular mechanisms underpinning its chaperone activity, and its implications in cellular physiology and pathology will be discussed below.

**Figure 2:**
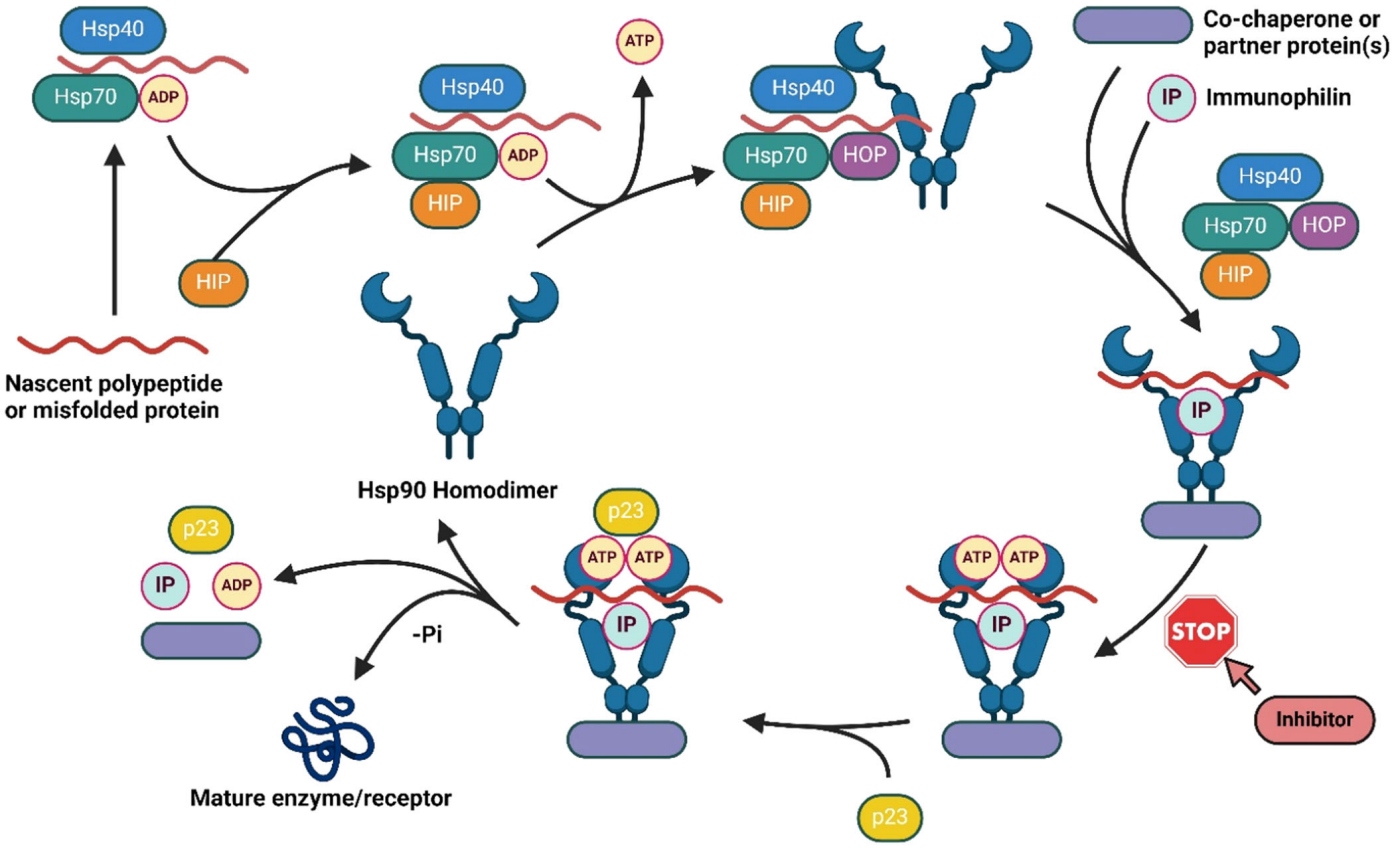
The protein folding cycle. [Color figure can be viewed at wileyonlinelibrary.com]

The chaperone cycle of Hsp90 (Figure 2) proceeds through a series of highly coordinated steps, initiated by the recognition and loading of client proteins onto the chaperone complex. ATP binding to the NTD signals a restructuring of the Hsp90‐Aha1 complex permitting NTD dimerization [59]. Co‐chaperones such as Hsp70 and HOP assist in client protein recognition and delivery to Hsp90, thereby initiating the folding process. Subsequent ATP hydrolysis drives conformational changes within the chaperone complex, facilitating client protein folding and maturation. The cycle culminates in the release of ADP and client proteins, followed by chaperone recycling for subsequent rounds of activity [22, 49].

Co‐chaperones play pivotal roles in modulating Hsp90's chaperone activity by regulating client protein recruitment, ATPase activity, and conformational dynamics. Hsp70 acts synergistically with Hsp90 during client protein folding, while co‐chaperones such as p23 and Aha1 fine‐tune Hsp90's ATPase activity and stabilizes client protein conformations [53, 54].

The dysregulation of Hsp90 chaperone activity has been implicated in various disease states, including cancer, neurodegenerative diseases, and metabolic disorders. Consequently, Hsp90 and its client proteins have emerged as a promising avenue for therapeutic intervention, with small molecule inhibitors and modulators of chaperone activity showing potential in preclinical and clinical settings [25].

## 3 Transcriptional Regulation of Hsp90α and Hsp90β

**Figure 3:**
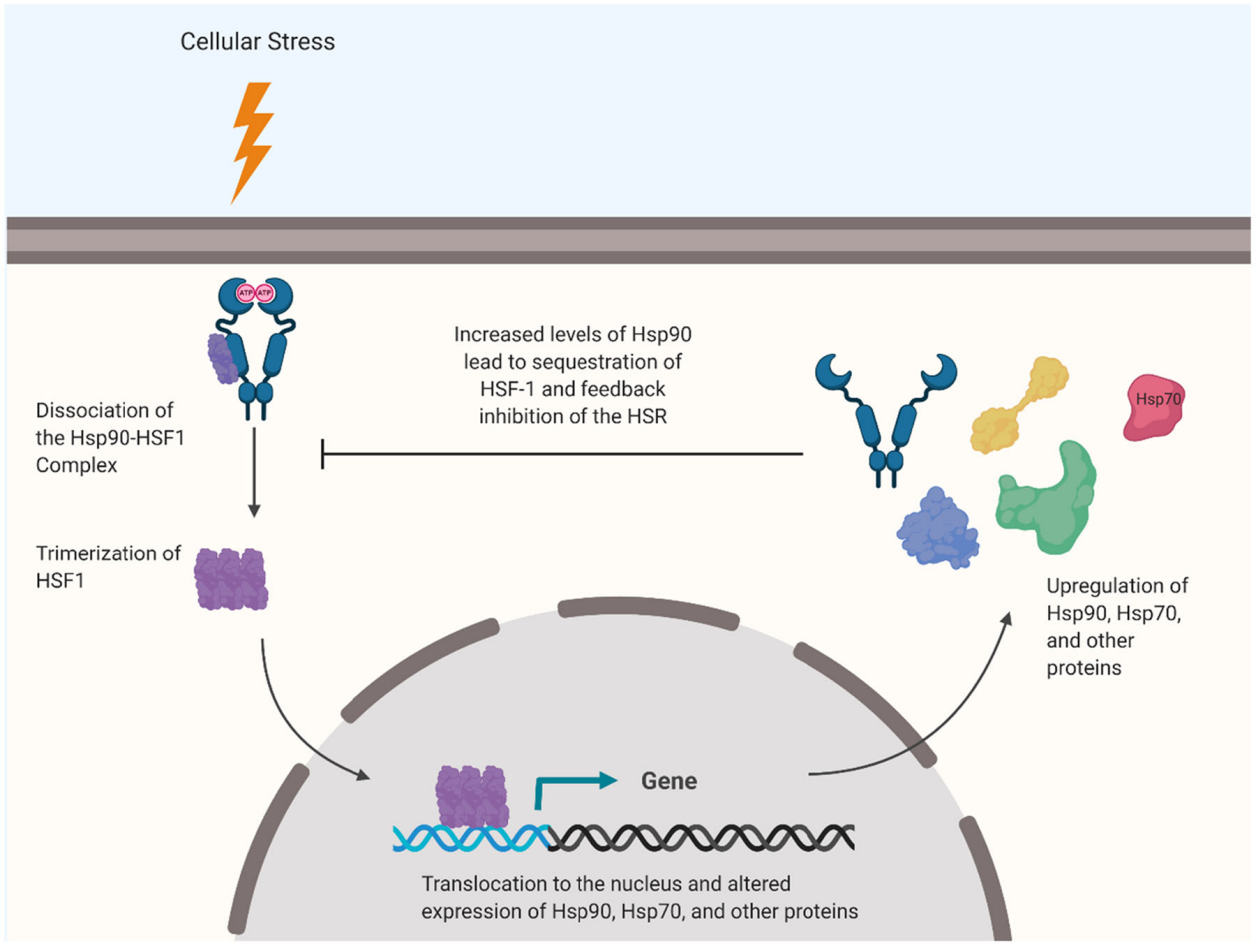
HSF1 Activation Cycle. [Color figure can be viewed at wileyonlinelibrary.com]

The transcriptional regulation of Heat Shock Proteins (HSPs), including Hsp90α and Hsp90β, is controlled by Heat Shock Elements (HSEs) (Figure 3). Heat Shock Factors (HSFs) are a group of transcriptional regulators that activate or repress transcription in both physiological and pathological conditions [48]. During cellular stress, HSFs are activated, binding to HSEs to modulate the transcription of HSP genes [60, 61, 62]. Specifically, Heat Shock Factor‐1 (HSF1) is the principal transcriptional regulator of Hsp90 and other HSPs. Under normal conditions, HSF1 remains inactive by forming a complex with Hsp90 and Hsp70 (Figure 2) [22].

Despite the functional diversity of Hsp90, there exists a high degree of structural identity between Hsp90α (HSP90AA1) and Hsp90β (HSP90AB1), with 86% amino acid sequence identity. This similarity reflects their evolution from a gene duplication event approximately 500 million years ago [16, 36, 63]. However, slight nucleotide sequence variations within noncoding regions allow differential regulation by interacting transcription factors. These variations result in subtle amino acid differences and unique Posttranslational modifications for each isoform, giving rise to their distinct regulatory patterns [43].

Research by Hickey and colleagues highlights that Hsp90α can be transcriptionally induced by heat or viral infection, although it is also constitutively expressed in various cell types. In contrast, Hsp90β, while expressed at lower levels during cellular stress, remains constitutively expressed and plays an essential role in the heat shock response (HSR) [5, 43]. Hsp90β is traditionally considered the “housekeeping” isoform, necessary for routine chaperone activities within cells, whereas Hsp90α is generally induced as a rapid response to environmental or pathological stressors [12].

Yufu and co‐workers demonstrated that Hsp90α is notably overexpressed in acute leukemia cells compared to normal blood cells, whereas Hsp90β expression remains low across both normal and diseased cells [64]. Notably, the overexpression of Hsp90α in leukemia patients occurs independently of drug treatment, suggesting that disease state, rather than treatment, induces Hsp90α. In contrast, Hsp90β remains at constitutive levels, reinforcing its role in baseline cellular function [46]. This dichotomy is intriguing, as it underscores the disease‐associated inducibility of Hsp90α while suggesting a more stable role for Hsp90β in routine cellular functions.

While Hsp90β has traditionally been considered a constitutively expressed isoform, more recent studies challenge this assumption by demonstrating context‐dependent inducibility. For example, an investigation by Neumann et al. found that specific immune cells, such as T lymphocytes, can upregulate Hsp90β in response to certain stimuli, indicating that its expression is not exclusively constitutive [46]. Other studies have similarly indicated inducibility under specific stress conditions. An in‐depth investigation by Kaji and colleagues showed that Hsp90β can be upregulated in particular cellular contexts, further challenging its strict classification as a housekeeping chaperone [65].

Furthermore, additional research has highlighted that Hsp90β is inducible in a subset of physiological and pathological situations, responding selectively to extracellular signals and changes in the cellular environment [66]. This context‐specific inducibility may be relevant to conditions requiring nuanced chaperone responses, where Hsp90β contributes uniquely to the stability and function of client proteins.

The selective inducibility of Hsp90β is not merely an isolated occurrence but has significant biological implications. Studies suggest that Hsp90β's inducibility in specific cell types, such as T lymphocytes, allows it to participate in immune responses, potentially facilitating the correct folding and stabilization of proteins critical to immune function [45]. This distinction between the isoforms is further supported by a review on Hsp90β's role, suggesting that while Hsp90α is predominantly involved in acute responses to cellular stress, Hsp90β provides a supportive role under stable, non‐stress conditions but can be upregulated to address specific cellular needs under certain conditions [67].

Such inducibility has broad implications, suggesting that Hsp90β may assist in maintaining cellular stability during prolonged or recurrent stress conditions, where constitutive chaperone activity may be insufficient. The ability of Hsp90β to be selectively induced could enhance cellular adaptability, supporting both protein homeostasis and the specific functional needs of various cell types.

Although high sequence homology between Hsp90α and Hsp90β complicates their biochemical differentiation, evidence increasingly suggests that these isoforms perform distinct functions in chaperoning client protein substrates. Hsp90α, highly inducible and responsive to cellular stress, rapidly mobilizes to address acute protein‐folding requirements. Hsp90β, in contrast, remains consistently involved in routine cellular processes but is capable of selective upregulation when required, reinforcing its role as a multifaceted chaperone suited to both constitutive and context‐dependent functions [16, 43].

This nuanced understanding of Hsp90β expands our appreciation of its role, highlighting that while it serves as a baseline chaperone under normal conditions, it can respond adaptively to cellular demands. This adaptive capacity aligns well with the need for fine‐tuned protein quality control, particularly in specialized cells or stress contexts where maintaining protein homeostasis is critical to cell survival.

## 4 Hsp90β‐Dependent Clients and Co‐Chaperones

Originally, it was believed that the two cytosolic isoforms exhibit redundant chaperone function and could compensate for the loss of one another [5]. However, growing experimental evidence suggests that each cytosolic isoform is responsible for discrete functions. Immunohistochemical investigations revealed that Hsp90β expressions and distributions are important at all stages of mouse embryo development, while Hsp90α was only noticed in diverse somatic cells [68]. Similarly experiments also demonstrated that lack of Hsp90β was critical to the development of placental labyrinth that resulted the death of mouse in Hsp90β knockout mouse models [69]. In addition, recent studies have shown that the co‐chaperones, FKBP8 and GCUNC45, exhibit preferential interaction with the β‐isoform [70, 71]. Interestingly, Hsp90α knockout mouse model shows that male mice without Hsp90α are apparently normal, including the development of their male reproductive system but cannot produce sperm [72]. Other experiments revealed that maturation and trafficking of the human *ether‐a‐gogo*‐related (hERG) protein is solely dependent upon the Hsp90α isoform [73]. These data indicate that both Hsp90 co‐chaperones and clients exhibit isoform preferences and/or isoform‐selective dependencies.

c‐IAP1 is a member of the inhibitor of apoptosis protein (IAP) family, which are responsible for cell differentiation, cell migration, immune response and most importantly, inhibition of apoptosis by suppressing caspase activity [74]. Experiments indicate that c‐IAP is dependent upon Hsp90β for its maturation, stability and translocation [75]. However, the same isoform is also involved in prolactin induced apoptosis in newt germ cells [76]. It appears that the prolactin receptor is an Hsp90β‐dependent client. CXCR_4_ and CDK‐6 have also been determined as Hsp90β‐dependent clients [77, 78].

The multi‐drug resistance (MDR) protein, P‐glycoprotein (PGP), is associated with the Hsp90β isoform [79]. Experiments with cancer cells have shown that Hsp90β is overexpressed in doxorubicin‐resistant cells as compared to cells that are doxorubicin sensitive [79]. Posttranslational modifications (PTMs) of Hsp90β significantly influence its activity, localization, and interaction with client proteins. Isoform‐specific phosphorylation on human Hsp90β modulated its binding affinity with the co‐chaperone Cdc37, enhancing its selectivity and stabilizing kinase clients. Phosphorylation at distinct serine and threonine residues on Hsp90β increased affinity toward Cdc37, making this interaction unique to Hsp90β compared to Hsp90α, where phosphorylation was either absent or less impactful. Additionally, Aha1, another co‐chaperone, bound more readily to Hsp90α than Hsp90β, suggesting that Aha1 played a more prominent role in modulating ATPase activity in Hsp90α, while Hsp90β was more finely tuned for kinase interactions through Cdc37. These findings highlighted functional distinctions between Hsp90α and Hsp90β, governed by phosphorylation patterns and client‐specific affinities [80, 81].

The stability of focal adhesion kinase (FAK), a signaling protein that is overexpressed in breast cancer, is directly dependent upon the Hsp90β isoform [82]. In addition, a host of cellular apoptosis regulatory proteins known as Bcl2 proteins have been linked to the Hsp90β isoform [83]. B cell mediated immune‐response, wherein CpG oligodeoxynucleotides (CpG ODNs) are activated, is also dependent upon the expression of Hsp90β [84]. Experiments with mouse phagocytes indicate an association between Bcl2 and Hsp90β. In addition, based on the structural and biochemical similarities between microtubule‐interacting protein (Mip‐90) and Hsp90β, it has been proposed that Hsp90β is required for cytoskeletal modulation [68, 85].

## 5 Extracellular Roles Played by Hsp90β

Since the discovery of Hsp90 as a potential target for several diseases, research has mostly been focused on intracellular Hsp90 and its chaperone function. However, recent studies have demonstrated extracellular Hsp90 (eHsp90) plays an important role, especially in tumorigenesis [28, 29, 30, 31, 32, 33]. It is unknown whether the eHsp90 is distinct from that of intracellular Hsp90 with regard to chaperone and substrate function [34]. There is experimental evidence that suggests eHsp90 consists of only the α‐isoform in human plasma and serum [34, 41]. In fact, elevated levels of eHsp90α have been observed in various cancer patients [86, 87]. Perhaps, eHsp90 is evolutionarily distinct from other isoforms, whereas Hsp90β solely located intracellularly and is involved in cellular development [34, 88]. This hypothesis suggests that eHsp90α is secreted during cellular stress; however, in cancer cells secreted Hsp90α appears to enhance cell migration during invasion and metastasis. If confirmed, extracellular Hsp90 could be selectively targeted to halt the disease progression.

Tumor suppressor p53 binds DNA and suppresses tumor cell proliferation. However, mutant p53 proteins are ineffective in their DNA binding activity and contribute to uncontrollable tumor progression [89, 90, 91]. Levine and co‐workers demonstrated that the application of a stressor, such as γ irradiation onto human non‐small‐cell lung cancer cells (H460) that contain wild type p53, led to the accumulation of eHsp90β (exosome) along with other secreted proteins. However, the H1299 cell line contains the mutated p53 allele and failed to secrete Hsp90β [92]. Together, these results indicate that the secretion of eHsp90β is likely a consequence of p53 activation, which may account for some of the observed inconsistencies.

Transforming growth factor‐β1 (TGF‐β1) is an important growth factor in embryonic development. Experiments with the MG63 osteosarcoma cell line demonstrated eHsp90β to negatively regulate cell proliferation via inhibition of the TGF‐β1 signaling cascade [93]. In the case of colon cancer (SW620 cell line), eHsp90β activated the αvβ6 mediated TGF‐β1 signaling pathway and enhanced tumor growth by increasing the migratory aptitude of these cells [94]. In contrast, experiments with human dermal and epi‐dermal cells showed that transforming growth factor α (TGFα) counters the effect of TGFβ1 and regulates the secretion of eHsp90α to promote re‐epithelialization at wound sites [40]. Correia and co‐workers showed that eHsp90β regulates matrix metalloproteinases 3 (MMP3), which is responsible for branching morphogenesis in mouse mammary epithelial cells [95]. Experiments with human retinal pigment epithelial cells (ARPE‐19 cells) showed that eHsp90β acts to induce inflammatory cascades by inducing the release of chemokines (IL‐8 and MCP‐1) [96]. These processes indicate a very distinct role for eHsp90β, which appears to be complementary to the roles played by intracellular Hsp90β.

## 6 Disease Implications for Hsp90β Inhibitors

The Hsp90 family controls the maturation of cellular and extracellular proteins such as kinases, growth factors, receptors, angiogenesis promoters, transcription factors, metalloproteinases, and telomerase amongst others [23] In addition, various mutated proteins are also dependent upon the Hsp90 machinery for chaperone and trafficking activity [97]. Not surprisingly, Hsp90 has emerged as a promising target to mitigate various pathological conditions during the last two decades [5, 11, 16, 17, 18, 19, 22, 23, 88].

Unfortunately, *pan*‐inhibition of all four isoforms (Hsp90α, Hsp90β, Grp94, and TRAP1) resulted in numerous unsuccessful drug discovery campaigns, which revealed dose‐limiting toxicities and induction of the pro‐survival heat shock response as the most common detriment [24, 25, 26, 27, 98, 99, 100]. As a result, it was proposed that the development of isoform‐selective inhibitors could overcome these detriments by reducing the number of substrates. Although isoform‐dependent clients of Hsp90 have not been fully elucidated, Hsp90β appears to represent a promising target that does not play a role in the cardio or ocular toxicities noted with *pan*‐inhibitors [78, 101].

### 6.1 Cancer

The Hsp90β isoform is an essential contributor to cell survival, promotion and growth in the early stages of life. For example, the Hsp90β isoform plays an important role in placenta development in mammals. In fact, phenotypic abnormalities were observed in genetically modified mice that expressed mutated Hsp90β [69]. Hsp90β‐linked transcriptional regulation also exhibited abnormalities during carcinogenesis, while Hsp90β showed aberrant activity in transformed cells [102]. Regardless of the high degree of homology, Hsp90α and Hsp90β exhibit distinct preferences for their substrate and co‐chaperones. Recently, selective inhibitors of each isoform were disclosed, and provide opportunities for the use of such inhibitors.

Experiments with leukemic cells showed the Hsp90β‐isoform was phosphorylated (except Ser 226 and Ser 255 residues) as compared to normal bone marrow cells in a Bcr‐Abl‐positive mouse model. Unphosphorylated Hsp90β was necessary to improve Apaf‐1 binding and induce cytochrome *c* mediated apoptosis [103]. It is well known that a host of human cancers are related to mutations in the tumor suppressor gene p53, which also associates with Hsp90 [104]. Isoform specific roles played by mutated p53 remain unknown, whereas the upregulation of p53 has been inversely correlated with Hsp90β expression. Immunoprecipitation experiments with mouse *myoblast* cells showed that Hsp90β preferentially binds to wild‐type p53, while Hsp90α preferentially binds mutated p53 [105].

A global health problem such as arsenic toxicity, which is responsible for many human diseases including cancer, has been linked to decreased levels of Hsp90β [106, 107, 108, 109]. Though it should be noted that arsenic toxicity is not dependent on any isoform of Hsp90. Experiments with glutathione‐deficient GCS‐2 cells exposed to arsenite toxins revealed that arsenites stabilize p53, which suppresses Hsp90β expression by binding its promotor [109]. Hsp90β levels could serve as a biomarker since elevated expression of this isoform is correlated with the size and severity of lung cancer [110, 111]. Hsp90β‐dependent growth of human endothelial cells is documented by experiments on human hepatocellular carcinoma in a tumor xenograft model [112]. Hsp90β is overexpressed in carcinogenic human endothelial cells that promoted the vascular endothelial growth factor receptors (VEGFRs) by increasing its promoter activity that are responsible for the creation of new blood vessels and tumor nourishment [112]. Similarly, the overexpression of Hsp90β was also detected in a human gastric cancer cell line (SGC7901/VCR of MDR‐type) when compared to normal SGCR901 cells [113]. While both isoforms are overexpressed in multiple myeloma (MM) cells, knockdown experiments demonstrated that unlike the α‐isoform, Hsp90β is indispensable for the survival of MM cells [114]. Experiments with colon cancer cells suggest Hsp90β to trigger an alternative αvβ6 integrin mediated TGF‐β1 signaling cascade as opposed to the canonical TGF‐β1 pathway [94]. Experiments with mouse mammary epithelial cells demonstrated Hsp90β to bind the hemopexin domain of Matrix metalloproteinases 3 (MMP3) whereas the inhibition of Hsp90β can prevent progression (invasion and branching) of the tumor [95]. It has also been proposed that Hsp90β can block the apoptotic functions of Bcl2 in Laryngeal carcinoma (LC) [83].

### 6.2 Viral Infections

Viruses are external pathogens responsible for infectious diseases. The isolated virus contains genetic material (DNA or RNA), a protein capsid and in some cases an outside envelope of lipids. After entering normal cells through various receptors, the virus hijacks the host machinery to replicate itself and invade neighboring cells throughout the host body [115]. At various stages of the virus life cycle, Hsp90 plays an important role [8, 116]. Viral infection induces the overexpression of Hsp90 due to its participation in cellular response mechanisms against infection [116]. Although the viral life cycle is mainly dependent upon the two cytosolic isoforms of Hsp90, reports on Hsp90 isoform function during viral infection are limited. However, Wang and co‐workers have provided a summary on the isoform specific role of Hsp90 during viral infection [117].

Enterovirus A71 (EV‐A71) is an epidemic virus that is responsible for neurological disorders, hand‐foot‐mouth disease in children and polio‐like conditions [118]. Experiments with a human rhabdomyosarcoma affected RD cell line revealed that the maturation and degradation of viral EV‐A71 capsid proteins are dependent upon the chaperone function of Hsp90β, although synthesis of the same viral polypeptide at the transcriptional level is not affected [119]. Interestingly, the downregulation of Hsp90β disrupted formation of a functional capsid protein and consequently, halted viral infection. Similarly, only Hsp90β has been shown to be associated with Japanese encephalitis virus (JEV)‐induced secretion proteins, which is required for viral infectivity [120]. In contrast, the porcine reproductive and respiratory syndrome virus (PRRSV), which is responsible for reproductive problems associated with sows and the corresponding respiratory disorder with piglets, is dependent upon both Hsp90α and Hsp90β for protein synthesis (N‐protein) at the transcriptional level [121]. Experiments on PRRSV host cells (MARC‐145 and PAM) demonstrated knockdown of both the α and β isoforms are required to reduce viral infection [122, 123, 124, 125].

### 6.3 Neurodegenerative Diseases

Hsp90 is deeply implicated in neurodegenerative diseases, where its role in providing neuroprotection is well‐documented through the degradation of denatured proteins. This function is particularly significant in diseases such as Alzheimer's (Aβ, tau), Parkinson's (α‐synuclein), and Huntington's (polyQ) [7, 126, 127]. Moreover, inhibiting Hsp90 can activate HSF‐1, the transcription factor and master regulator of the heat shock response (HSR). Inhibition of cytosolic Hsp90 disrupts the complex state of HSF‐1, promoting its trimer formation and translocation into the nucleus. Therein, it facilitates the transcription of Hsp90, Hsp70, Hsp40, and Hsp27 offering neuroprotection against various neurotoxic insults [5, 126]. While the selective modulation of Hsp90 isoforms to alleviate neurodegenerative diseases is still being explored, intriguing reports suggest that isoform selectivity may play a pivotal role in neuroprotection.

A site‐selective approach targeting the Hsp90 N‐terminal binding pocket, specifically for cytosolic isoforms α and β over Grp94 and TRAP‐1, has been emphasized in Huntington's disease (HD) [18, 128]. Through in‐depth analysis supported by X‐ray crystal structures, it has been revealed that inhibitors targeting site‐1 (residues 104–111) of the ATP‐binding pocket induce a new α‐helical conformation, which stabilizes and shields this region from other interactions. Such observations enabled the design of Hsp90 α/β isoform‐selective inhibitors, and significantly reduced the amyloidogenic mutant huntingtin (mHTT) aggregates responsible for HD pathogenicity, while mitigating toxicity associated with pan‐inhibition [128, 129].

The association between multiple sclerosis (MS) pathogenesis and Hsp90β has been suggested by the presence of anti‐Hsp90β antibodies in the cerebrospinal fluid (CSF) of MS patients [130]. In Alzheimer's disease, the downregulation of peroxisome proliferator‐activated receptor γ (PPARγ) and the accumulation of Aβ42 peptides in the brain correlate with a decrease in Hsp90β levels in BV2 cells in mouse models, while Hsp90α expression remains unaltered, indicating distinct roles for the two isoforms [131]. Conversely, Hsp90α levels are shown to increase during Alzheimer's inflammation [132, 133]. Targeting Hsp90α and its co‐chaperone and ATPase activator Aha1 with inhibitors, such as KU‐177, reduces tau aggregations in Alzheimer's disease [134, 135]. In addition, extracellular Hsp90 isoforms and their family members (Hsp40, Hsp70, and Hsp110) secreted from non‐neuronal cells in the extracellular environment also serve as a response to neurodegeneration and neuro‐abnormalities associated with aging [127].

### 6.4 Other Diseases and Applications

Cytosolic Hsp90 has been implicated in a wide array of diseases, disorders, and inflammatory conditions, including cystic fibrosis (CFTR), atherosclerosis, diabetes, and metabolic disorders. However, recent research has underscored the importance of considering isoform‐specific roles in these contexts [5].

It has been proposed that Hsp90β‐selective inhibitors could serve as a drug target for treating patients with metabolic disorders such as obesity‐induced fatty liver disease, type 2 diabetes, and atherosclerosis. Studies have revealed that Hsp90β, rather than Hsp90α, is notably overexpressed in the hepatocytes of individuals with nonalcoholic fatty liver disease (NAFLD) and diet‐induced obese (DIO) mice [136]. Further experiments conducted on obese children (median age 10) corroborated these findings, indicating a significant elevation of Hsp90β levels when compared to Hsp90α levels, particularly upon transitioning from non‐NAFLD to NAFLD cases. This suggests that the Hsp90α/β ratio could potentially serve as a biomarker for NAFLD and related disorders [137]. Notably, both isoforms have been implicated in wound healing, albeit with distinct, nonoverlapping roles.

Intracellularly, Hsp90β functions to stabilize the lipoprotein receptor‐related protein‐1 (LRP‐1), which is located at the cell membrane, whereas Hsp90 operates extracellularly, promoting cell mortality at wound sites [138]. Higher levels of Hsp90β expression are associated with cellular inflammation and organ injury [139]. In cases of gastric disorders, inflammation is mediated by the activation of NADPH‐oxidase in gastric epithelial cells. Experimental evidence suggests that the G protein, Rac1, a component of the NADPH oxidase complex, interacts directly with membrane‐translocated Hsp90β, facilitating NADPH‐oxidase‐mediated reactive oxygen species (ROS) formation in H. pylori‐infected AGS cells (gastric epithelial cells). Conversely, downregulation of Hsp90β has been shown to reduce H_2_O_2_ levels in the same cell lines, suggesting that the inhibition of Hsp90β may hold promise for mitigating gastric disorders [39].

Use of Hsp90β inhibitors has demonstrated enhanced antinociceptive effects of morphine in mice by activating the ERK‐RSK pathway, specifically within the spinal cord exclusive of the brain and periphery [140, 141, 142]. The underlying activity was subsequently refined as resulting from suppression of an AMPK‐mediated negative feedback loop [143]. Whereas activation of ERK enhanced antinociceptive activity, activation of AMPK suppresses ERK. It's noteworthy that this enhanced efficacy of morphine comes without the undesirable increase in tolerance and constipation. Not exclusive to the spinal cord, it was recently reported that selective inhibitors of Hsp90β significantly decreased lipopolysaccharide (LPS)‐induced production of inflammatory mediators (NO, IL‐1β, and TNF‐α) in murine microglial BV‐2 cells by attenuating NF‐κB and ERK MAPK activation [144]. In contrast, inhibitors targeting Hsp90α, Grp94, and TRAP1 had minimal impact on these mediators, highlighting Hsp90β as central to LPS‐induced neuroinflammation and represents a promising drug target for developing pain medications with potentially fewer adverse effects compared to pan‐Hsp90 inhibitors [144].

## 7 Hsp90β‐Selective Inhibition

Given the close similarities between members of the Hsp90 family, especially Hsp90α and Hsp90β, isoform‐selective inhibition is not without its challenges. The binding pocket is 85% identical across all four isoforms, and there is > 95% identity between the ATP‐binding pocket of Hsp90α and Hsp90β; differing by only two amino acids with Hsp90β containing Ala52 and Leu91 in lieu of Ser52 and Ile91 for Hsp90α (numbering as per PDB code 1UYM). Despite this shared identity, there are sufficient differences that can be exploited to selectively inhibit each isoform and avoid the associated risks of *pan*‐inhibition [145, 146, 147].

**Figure 4:**
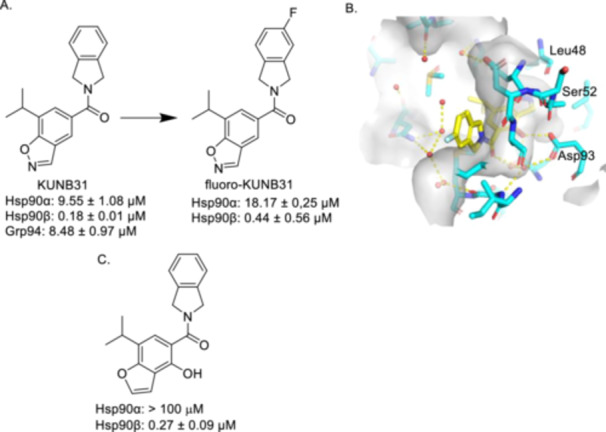
A. Structure and affinity of KUNB31 and fluoro‐KUNB31. B. Co‐crystal structure of fluoro‐KUNB31 bound to Hsp90β via a network of water molecules (PDB Code: 5UCJ). C. Structure and selectivity of benzofuran analog of KUNB31 [101]. [Color figure can be viewed at wileyonlinelibrary.com]

The first Hsp90β‐selective inhibitor, KUNB31 (Figure 4), manifested an IC_50_ value via FP assay of 0.18 ± 0.01 µM for Hsp90β versus 9.55 ± 1.08 µM and 8.48 ± 0.97 µM for Hsp90α and Grp94, respectively [78]. The antiproliferative potential of KUNB31 was also assessed against various cancer cell lines, including NCI H23 (non‐small cell lung cancer), UC3 (bladder cancer), and HT‐29 (colon adenocarcinoma), alongside noncancerous HEK 293 (human embryonic kidney) cells. KUNB31 demonstrated good activity, with IC_50_ values of 6.74 ± 1.10 µM, 3.01 ± 0.56 µM, and 3.72 ± 0.34 µM against NCI H23, UC3, and HT‐29 cancer cell lines, respectively, while requiring concentrations in excess of 100 µM against HEK‐293 cells, demonstrating a high differential selectivity. Most importantly, and in contrast to pan‐Hsp90 inhibitors, KUNB31 did not induce the upregulation of Hsp90 levels, nor did it induce HSF1 activation. Subsequent SAR studies identified fluoro‐KUNB31 (Figure 4) with comparable selectivity with a slight loss of affinity [148, 149]. Extended SAR studies on KUNB31 like scaffolds resulted in a benzofuran analog of KUNB31 (Structure C, Figure 4) that showed much improved selectivity, however, this compound failed to display similar potency when tested against various cancer cell lines [101].

**Figure 5:**
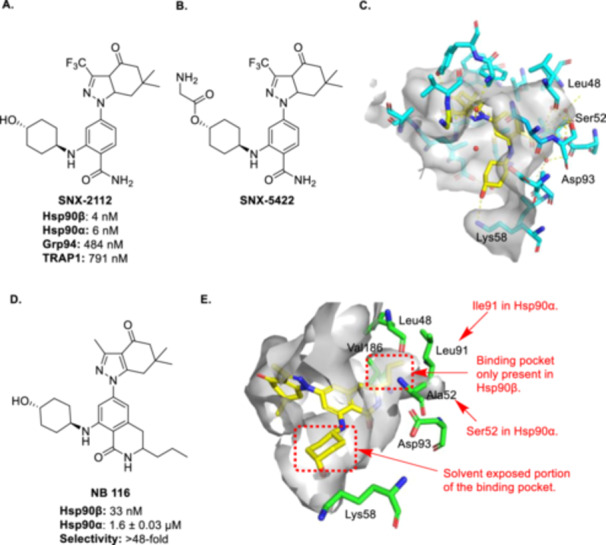
(A) Structure and affinity of SNX‐2112. (B) Structure of SNX‐5422 (prodrug of SNX‐2112. (C) SNX‐2112 docked in Hsp90α. (PDB Code: 6LTK) (D) Structure and affinity of NB 116 as determined by FP assay. (E) Proposed binding mode of NB 116 in the Hsp90β binding site (PDB Code: 1UYM). [Color figure can be viewed at wileyonlinelibrary.com]

NB 116 (compound 5a in source paper) was subsequently disclosed and related to SNX‐2112; a *pan*‐inhibitor that demonstrated good inhibitory activity, but whose clinical trials were terminated due to ocular toxicity [150, 151, 152]. SNX‐2112 (Figure 5A) and its orally available prodrug, SNX‐5422 (Figure 5B), facilitated interactions with conserved water molecules to enhance occupation of the ATP‐binding site as shown in Figure 5C.

NB 116 was designed to decrease the entropic penalty paid upon binding, while simultaneously occupying a unique binding pocket that exists solely in Hsp90β and exhibited a 48‐fold increase in affinity for Hsp90β over Hsp90α with an IC_50_ value of 33 nM (Figure 5D) [150]. This selectivity was achieved by exploitation of a small pocket specific to Hsp90β formed by Val186, Leu48, Leu91, Ala52, and Asp93 (Figure 5E). Projection of the propyl moiety of NB 116 into this pocket provides enhanced affinity and selectivity for Hsp90β [153]. Furthermore, replacement of the propyl group with a butyl group increased selectivity for Hsp90β over Hsp90α, but at the expense of affinity (Hsp90β IC_50_ = 0.156 μM; Hsp90α IC_50_ > 50 μM; Grp94 IC_50_ > 5 μM; TRAP1 IC_50_ > 5 μM).

**Figure 6:**
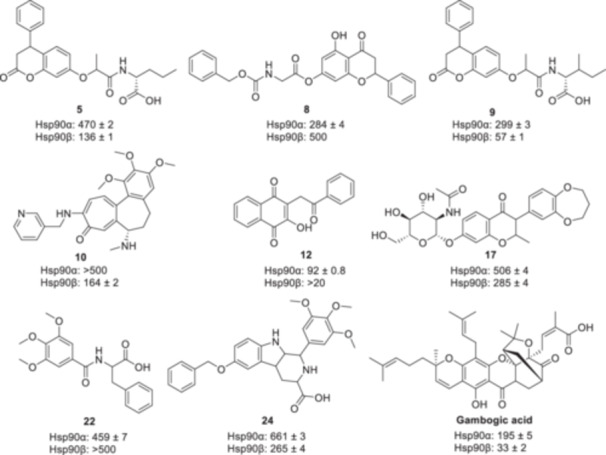
MD inhibitors to Hsp90α/β and associated K_D_ values (µM). Gambogic acid was used as a control.

A series of Hsp90β‐selective inhibitors that bind the MD were recently reported after a virtual screen of a library of 9,051 natural products (Figure 6) [154]. These lead compounds could serve as the basis for continued SAR studies to enhance affinity while maintaining or improving selectivity.

While research on the development of Hsp90 inhibitors has been pursued extensively over the past decades, the development of isoform‐specific inhibitors is new and represents the current frontier of Hsp90 modulation.

## 8 Conclusion

The Hsp90β isoform represents a promising and attractive target for drug development due to its constitutive expression and unique role in cellular homeostasis. Unlike its inducible counterpart Hsp90α, Hsp90β's involvement in fundamental cellular processes and disease mechanisms, particularly in cancer and neurodegenerative disorders, highlights its therapeutic potential. The challenges faced with pan‐Hsp90 inhibitors, including adverse effects and toxicity, emphasize the need for isoform‐specific inhibitors. By targeting Hsp90β selectively, there is a potential to mitigate these issues and develop more effective and safer therapeutic interventions. Continued research into the structural and functional distinctions of Hsp90β will be crucial in advancing drug discovery efforts and unlocking new opportunities for the treatment of various diseases.

## Conflicts of Interest

Brian S.J. Blagg is a cofounder of Grannus Therapeutics.

## Data Availability

The data that support the findings of this study are available on request from the corresponding author. The data are not publicly available due to privacy or ethical restrictions.
